# Tetra­kis[μ-4-(methyl­amino)­benzoato-κ^2^ *O*:*O*′]bis­[(*N*,*N*-diethyl­nicotinamide-*N* ^1^)zinc(II)] dihydrate

**DOI:** 10.1107/S1600536809040409

**Published:** 2009-10-10

**Authors:** Tuncer Hökelek, Filiz Yılmaz, Barış Tercan, Özgür Aybirdi, Hacali Necefoğlu

**Affiliations:** a Department of Physics, Hacettepe University, 06800 Beytepe, Ankara, Turkey; b Department of Physics, Faculty of Science, Anadolu University, Department of Chemistry, 26470 Yenibağlar, Eskişehir, Turkey; c Department of Physics, Karabük University, 78050 Karabük, Turkey; d Department of Chemistry, Kafkas University, 63100 Kars, Turkey

## Abstract

The title mol­ecule, [Zn_2_(C_8_H_8_NO_2_)_4_(C_10_H_14_N_2_O)_2_]·2H_2_O, is a centrosymmetric binuclear complex, with two Zn^II^ ions [Zn⋯Zn’ = 2.9301 (4) Å] bridged by four methyl­amino­benzoate (MAB) ligands. The four nearest O atoms around each Zn^II^ ion form a distorted square-planar arrangement with the distorted square-pyramidal coordination completed by the pyridine N atom of the *N*,*N*-diethyl­nicotinamide (DENA) ligand. Each Zn^II^ ion is displaced by 0.3519 (2) Å from the plane of the four O atoms, with an average Zn—O distance of 2.030 Å. The dihedral angles between carboxyl­ate groups and adjacent benzene rings are 10.57 (10) and 16.63 (12)°, while the benzene rings are oriented at a dihedral angle of 81.84 (5)°. The pyridine ring is oriented at dihedral angles of 40.49 (6) and 51.25 (6)° with respect to the benzene rings. In the crystal structure, inter­molecular O—H⋯O and N—H⋯O hydrogen bonds link the mol­ecules into a three-dimensional network. The π–π contact between the inversion-related pyridine rings [centroid–centroid distance = 3.633 (1) Å] may further stabilize the crystal structure.

## Related literature

For niacin, see: Krishnamachari (1974 ▶) and for the nicotinic acid derivative *N*,*N*-diethyl­nicotinamide, see: Bigoli *et al.* (1972 ▶). For related structures, see: Hökelek *et al.* (1995 ▶, 2009 ▶); Speier & Fulop (1989 ▶); Usubaliev *et al.* (1980 ▶).
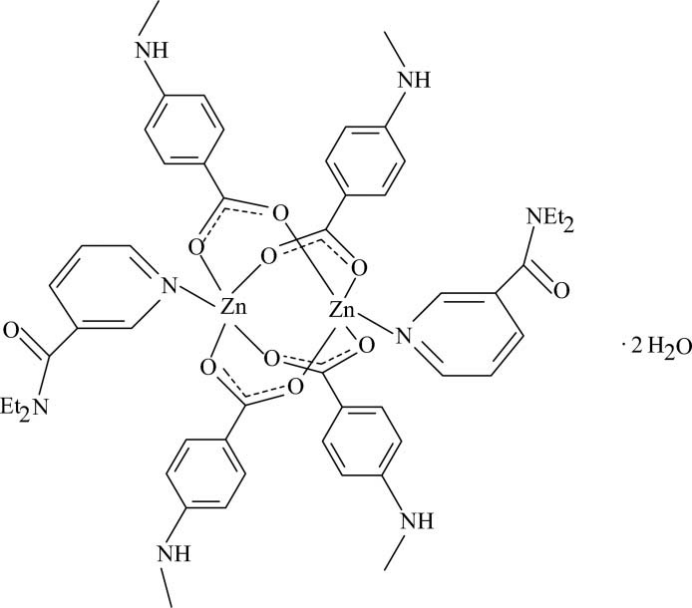


## Experimental

### 

#### Crystal data

[Zn_2_(C_8_H_8_NO_2_)_4_(C_10_H_14_N_2_O)_2_]·2H_2_O
*M* *_r_* = 1123.89
Monoclinic, 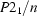

*a* = 10.1384 (2) Å
*b* = 25.6931 (3) Å
*c* = 10.5170 (4) Å
β = 103.061 (2)°
*V* = 2668.67 (12) Å^3^
*Z* = 2
Mo *K*α radiation
μ = 0.97 mm^−1^
*T* = 100 K
0.52 × 0.52 × 0.10 mm

#### Data collection

Bruker APEXII CCD area-detector diffractometer
Absorption correction: multi-scan (*SADABS*; Bruker, 2005 ▶) *T*_min_ = 0.609, *T*_max_ = 0.905
23512 measured reflections
6638 independent reflections
5562 reflections with *I* > 2σ(*I*)
*R*_int_ = 0.029

#### Refinement

*R*[*F* ^2^ > 2σ(*F* ^2^)] = 0.035
*wR*(*F* ^2^) = 0.086
*S* = 1.02
6638 reflections
346 parameters
H atoms treated by a mixture of independent and constrained refinement
Δρ_max_ = 0.74 e Å^−3^
Δρ_min_ = −0.54 e Å^−3^

### 

Data collection: *APEX2* (Bruker, 2007 ▶); cell refinement: *SAINT* (Bruker, 2007 ▶); data reduction: *SAINT*; program(s) used to solve structure: *SHELXS97* (Sheldrick, 2008 ▶); program(s) used to refine structure: *SHELXL97* (Sheldrick, 2008 ▶); molecular graphics: *ORTEP-3 for Windows* (Farrugia, 1997 ▶); software used to prepare material for publication: *WinGX* (Farrugia, 1999 ▶) and *PLATON* (Spek, 2009 ▶).

## Supplementary Material

Crystal structure: contains datablocks I, global. DOI: 10.1107/S1600536809040409/ci2912sup1.cif

Structure factors: contains datablocks I. DOI: 10.1107/S1600536809040409/ci2912Isup2.hkl

Additional supplementary materials:  crystallographic information; 3D view; checkCIF report

## Figures and Tables

**Table 1:** Selected bond lengths (Å)

Zn1—O1	2.0224 (13)
Zn1—O2	2.0207 (13)
Zn1—O3	2.0819 (14)
Zn1—O4	2.0459 (13)
Zn1—N1	2.0516 (14)

**Table 2:** Hydrogen-bond geometry (Å, °)

*D*—H⋯*A*	*D*—H	H⋯*A*	*D*⋯*A*	*D*—H⋯*A*
N3—H3*A*⋯O6^i^	0.86	2.50	3.105 (3)	128
N4—H4*A*⋯O6^ii^	0.86	2.07	2.922 (3)	171
O6—H61⋯O4^iii^	0.93 (4)	2.07 (4)	2.875 (2)	143 (3)
O6—H61⋯O2^iii^	0.93 (4)	2.37 (4)	3.117 (2)	137 (3)
O6—H62⋯O5^iv^	0.96 (4)	1.81 (4)	2.741 (2)	162 (3)

Symmetry codes: (i) 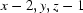; (ii) 
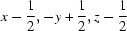
; (iii) 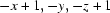; (iv) 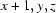.

**Fig. 1.**
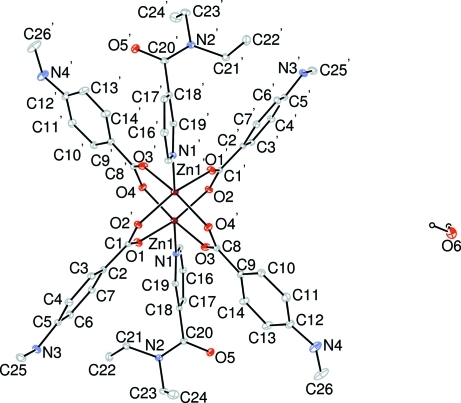
The molecular structure of the title compound, with the atom-numbering scheme. Displacement ellipsoids are drawn at the 30% probability level. H atoms have been omitted for clarity. Primed atoms are generated by the symmetry operator: (') 1 - x, -y, -z.

## supplementary crystallographic information

### Comment

As a part of our ongoing investigation on transition metal complexes of nicotinamide (NA), one form of niacin (Krishnamachari, 1974), and/or the nicotinic acid derivative *N*,*N*-diethylnicotinamide (DENA), an important respiratory stimulant (Bigoli *et al.*, 1972), the title compound was synthesized and its crystal structure is reported herein.

The title compound is a binuclear compound, consisting of two DENA and four methylaminobenzoate (MAB) ligands. The crystal structures of similar complexes of Cu^2+^ and Zn^2+^ ions, [Cu(C_6_H_5_COO)_2_(C_5_H_5_N)]_2_ (Usubaliev *et al.*, 1980), [Cu(C_6_H_5_CO_2_)_2_(py)]_2_ (Speier & Fulop, 1989), [Cu_2_(C_6_H_5_COO)_4_(C_10_H_14_N_2_O)_2_] (Hökelek *et al.*, 1995) and [Zn_2_(C_11_H_14_NO_2_)_4_(C_10_H_14_N_2_O)_2_] (Hökelek *et al.*, 2009) have also been reported. In these structures, the benzoate ion acts as a bidentate ligand.

The title dimeric complex, [Zn_2_(MAB)_4_(DENA)_2_].H_2_O, has a centre of symmetry and the two Zn^II^ ions are surrounded by four MAB groups and two DENA ligands. The DENA ligands are coordinated to Zn^II^ ions through pyridine N atoms only. The MAB groups act as bridging ligands. The Zn···Zn' distance is 2.9301 (4) Å. The average Zn—O distance is 2.0297 (13) Å (Table 1), and four O atoms of the bridging MAB ligands around each Zn^II^ ion form a distorted square plane. The Zn^II^ ion lies 0.3519 (2) Å below the least-squares plane. The average O—Zn—O bond angle is 88.30 (6)°. A distorted square-pyramidal arrangement around each Zn^II^ ion is completed by the pyridine N atom of DENA ligand at 2.0516 (14) Å (Table 1) from the Zn atom. The N1—Zn1···Zn1' angle is 159.67 (4)° and the dihedral angle between plane through atoms Zn1, O1, O2, C1, Zn1', O1', O2'and C1' and the plane through Zn1, O3, O4, C8, Zn1', O3', O4', and C8' atoms is 89.00 (7)°. The dihedral angles between the planar carboxylate groups and the adjacent benzene rings A (C2—C7) and B (C9—C14) are 10.57 (10)° and 16.63 (12)°, respectively, while that between rings A and B is A/B = 81.84 (5)°. Ring C (N1/C15-C19) is oriented with respect to rings A and B at dihedral angles A/C = 40.49 (6) and B/C = 51.25 (6) °.

In the crystal structure, intermolecular O—H···O and N—H···O interactions (Table 2) link the molecules into a three-dimensional network, in which they may be effective in the stabilization of the structure. The π–π contact between the pyridine rings, *Cg*1—*Cg*1^i^, [symmetry code (i): 2 - *x*, -*y*, 1 - *z*, where *Cg*1 is centroid of the ring C (N1/C15—C19)] may further stabilize the structure, with centroid-centroid distance of 3.633 (1) Å.

### Experimental

The title compound was prepared by the reaction of ZnSO_4_.H_2_O (0.9 g, 5 mmol) in H_2_O (30 ml) and DENA (1.78 g, 10 mmol) in H_2_O (20 ml) with sodium *p*-methylaminobenzoate (1.74 g, 10 mmol) in H_2_O (50 ml). The mixture was filtered and set aside to crystallize at ambient temperature for one week, giving colourless single crystals.

### Refinement

Atoms H61 and H62 (for H_2_O) were located in a difference Fourier map and refined isotropically. The remaining H atoms were positioned geometrically with N-H = 0.86 Å and C-H = 0.93, 0.97 and 0.96 Å, for aromatic, methylene and methyl H atoms, respectively, and constrained to ride on their parent atoms, with *U*_iso_(H) = *xU*_eq_(C,*N*), where *x* = 1.5 for methyl H and *x* = 1.2 for all other H atoms.

### Crystal data

**Table:**

[Zn_2_(C_8_H_8_NO_2_)_4_(C_10_H_14_N_2_O)_2_]·2H_2_O	*F*(000) = 1176
*M**_r_* = 1123.89	*D*_x_ = 1.399 Mg m^−^^3^
Monoclinic, *P*2_1_/*n*	Mo *K*α radiation, λ = 0.71073 Å
Hall symbol: -P 2yn	Cell parameters from 8651 reflections
*a* = 10.1384 (2) Å	θ = 3.0–28.2°
*b* = 25.6931 (3) Å	µ = 0.97 mm^−^^1^
*c* = 10.5170 (4) Å	*T* = 100 K
β = 103.061 (2)°	Plate, colorless
*V* = 2668.67 (12) Å^3^	0.52 × 0.52 × 0.10 mm
*Z* = 2	

### Data collection

**Table:**

Bruker Kappa APEXII CCD area-detector diffractometer	6638 independent reflections
Radiation source: fine-focus sealed tube	5562 reflections with *I* > 2σ(*I*)
graphite	*R*_int_ = 0.029
φ and ω scans	θ_max_ = 28.3°, θ_min_ = 1.6°
Absorption correction: multi-scan (*SADABS*; Bruker, 2005)	*h* = −13→10
*T*_min_ = 0.609, *T*_max_ = 0.905	*k* = −30→34
23512 measured reflections	*l* = −14→14

### Refinement

**Table:**

Refinement on *F*^2^	Primary atom site location: structure-invariant direct methods
Least-squares matrix: full	Secondary atom site location: difference Fourier map
*R*[*F*^2^ > 2σ(*F*^2^)] = 0.035	Hydrogen site location: inferred from neighbouring sites
*wR*(*F*^2^) = 0.086	H atoms treated by a mixture of independent and constrained refinement
*S* = 1.02	*w* = 1/[σ^2^(*F*_o_^2^) + (0.0383*P*)^2^ + 2.063*P*] where *P* = (*F*_o_^2^ + 2*F*_c_^2^)/3
6638 reflections	(Δ/σ)_max_ = 0.001
346 parameters	Δρ_max_ = 0.74 e Å^−^^3^
0 restraints	Δρ_min_ = −0.54 e Å^−^^3^

### Special details

**Table:**

Geometry. All e.s.d.'s (except the e.s.d. in the dihedral angle between two l.s. planes) are estimated using the full covariance matrix. The cell e.s.d.'s are taken into account individually in the estimation of e.s.d.'s in distances, angles and torsion angles; correlations between e.s.d.'s in cell parameters are only used when they are defined by crystal symmetry. An approximate (isotropic) treatment of cell e.s.d.'s is used for estimating e.s.d.'s involving l.s. planes.
Refinement. Refinement of *F*^2^ against ALL reflections. The weighted *R*-factor *wR* and goodness of fit *S* are based on *F*^2^, conventional *R*-factors *R* are based on *F*, with *F* set to zero for negative *F*^2^. The threshold expression of *F*^2^ > σ(*F*^2^) is used only for calculating *R*-factors(gt) *etc*. and is not relevant to the choice of reflections for refinement. *R*-factors based on *F*^2^ are statistically about twice as large as those based on *F*, and *R*- factors based on ALL data will be even larger.

### Fractional atomic coordinates and isotropic or equivalent isotropic displacement parameters (Å^2^)

**Table:**

	*x*	*y*	*z*	*U*_iso_*/*U*_eq_	
Zn1	−0.04338 (2)	−0.014479 (8)	0.118981 (18)	0.01584 (6)	
O1	−0.20843 (14)	0.01538 (5)	−0.00434 (12)	0.0240 (3)	
O2	0.14508 (13)	−0.04361 (5)	0.18309 (12)	0.0202 (3)	
O3	0.03250 (15)	0.06111 (5)	0.13786 (14)	0.0268 (3)	
O4	−0.09993 (14)	−0.08625 (5)	0.04128 (12)	0.0225 (3)	
O5	−0.19322 (15)	0.13404 (5)	0.50417 (14)	0.0281 (3)	
O6	0.95980 (18)	0.15202 (7)	0.75046 (18)	0.0397 (4)	
H61	0.967 (4)	0.1239 (15)	0.808 (4)	0.087 (12)*	
H62	0.904 (4)	0.1385 (15)	0.671 (4)	0.088 (13)*	
N1	−0.12123 (15)	−0.00735 (6)	0.28204 (14)	0.0166 (3)	
N2	−0.39214 (16)	0.12268 (6)	0.35808 (15)	0.0201 (3)	
N3	−0.73560 (17)	0.14248 (7)	−0.25237 (16)	0.0279 (4)	
H3A	−0.7889	0.1404	−0.1995	0.033*	
N4	0.2505 (2)	0.28515 (7)	0.3274 (2)	0.0434 (5)	
H4A	0.3067	0.3035	0.2962	0.052*	
C1	−0.23030 (18)	0.03966 (7)	−0.11144 (16)	0.0168 (3)	
C2	−0.36417 (18)	0.06493 (7)	−0.15463 (16)	0.0162 (3)	
C3	−0.39620 (19)	0.09599 (7)	−0.26573 (17)	0.0194 (4)	
H3	−0.3338	0.0997	−0.3178	0.023*	
C4	−0.5190 (2)	0.12146 (7)	−0.29995 (17)	0.0217 (4)	
H4	−0.5382	0.1421	−0.3744	0.026*	
C5	−0.61546 (19)	0.11639 (7)	−0.22320 (17)	0.0204 (4)	
C6	−0.58436 (19)	0.08385 (7)	−0.11255 (18)	0.0217 (4)	
H6	−0.6477	0.0789	−0.0620	0.026*	
C7	−0.46113 (19)	0.05944 (7)	−0.07914 (17)	0.0194 (4)	
H7	−0.4413	0.0388	−0.0047	0.023*	
C8	0.08144 (18)	0.09445 (7)	0.07307 (17)	0.0196 (4)	
C9	0.1199 (2)	0.14569 (7)	0.13448 (17)	0.0212 (4)	
C10	0.2042 (2)	0.17976 (7)	0.0866 (2)	0.0248 (4)	
H10	0.2328	0.1713	0.0112	0.030*	
C11	0.2458 (2)	0.22608 (8)	0.1502 (2)	0.0298 (4)	
H11	0.3032	0.2481	0.1178	0.036*	
C12	0.2017 (2)	0.24024 (8)	0.2638 (2)	0.0304 (5)	
C13	0.1107 (2)	0.20754 (8)	0.3067 (2)	0.0321 (5)	
H13	0.0755	0.2173	0.3776	0.038*	
C14	0.0728 (2)	0.16080 (8)	0.24425 (19)	0.0274 (4)	
H14	0.0147	0.1388	0.2758	0.033*	
C15	−0.10860 (18)	−0.04226 (7)	0.37916 (16)	0.0170 (3)	
H15	−0.0671	−0.0740	0.3710	0.020*	
C16	−0.15508 (19)	−0.03269 (7)	0.49092 (17)	0.0195 (4)	
H16	−0.1474	−0.0580	0.5555	0.023*	
C17	−0.21321 (18)	0.01504 (7)	0.50527 (17)	0.0189 (3)	
H17	−0.2428	0.0227	0.5807	0.023*	
C18	−0.22681 (18)	0.05145 (7)	0.40509 (16)	0.0166 (3)	
C19	−0.18061 (18)	0.03849 (7)	0.29495 (16)	0.0174 (3)	
H19	−0.1911	0.0625	0.2272	0.021*	
C20	−0.27156 (19)	0.10611 (7)	0.42525 (17)	0.0190 (4)	
C21	−0.4910 (2)	0.09017 (8)	0.26852 (18)	0.0238 (4)	
H21A	−0.4441	0.0615	0.2379	0.029*	
H21B	−0.5342	0.1108	0.1933	0.029*	
C22	−0.5985 (2)	0.06864 (9)	0.3335 (2)	0.0320 (5)	
H22A	−0.6640	0.0495	0.2707	0.048*	
H22B	−0.6426	0.0968	0.3672	0.048*	
H22C	−0.5570	0.0460	0.4037	0.048*	
C23	−0.4330 (2)	0.17653 (7)	0.3789 (2)	0.0264 (4)	
H23A	−0.3975	0.1861	0.4695	0.032*	
H23B	−0.5310	0.1784	0.3616	0.032*	
C24	−0.3819 (3)	0.21482 (9)	0.2916 (3)	0.0398 (6)	
H24A	−0.4139	0.2491	0.3050	0.060*	
H24B	−0.4147	0.2049	0.2020	0.060*	
H24C	−0.2847	0.2147	0.3124	0.060*	
C25	−0.7761 (2)	0.17342 (9)	−0.3688 (2)	0.0345 (5)	
H25A	−0.8664	0.1862	−0.3752	0.052*	
H25B	−0.7740	0.1524	−0.4438	0.052*	
H25C	−0.7151	0.2022	−0.3650	0.052*	
C26	0.2125 (4)	0.30333 (9)	0.4443 (2)	0.0561 (8)	
H26A	0.2589	0.3353	0.4726	0.084*	
H26B	0.2368	0.2776	0.5118	0.084*	
H26C	0.1165	0.3091	0.4261	0.084*	

### Atomic displacement parameters (Å^2^)

**Table:**

	*U*^11^	*U*^22^	*U*^33^	*U*^12^	*U*^13^	*U*^23^
Zn1	0.01825 (11)	0.01770 (11)	0.01276 (10)	0.00284 (8)	0.00601 (7)	0.00051 (7)
O1	0.0237 (7)	0.0314 (7)	0.0170 (6)	0.0086 (6)	0.0049 (5)	0.0050 (5)
O2	0.0183 (6)	0.0244 (7)	0.0186 (6)	0.0045 (5)	0.0054 (5)	0.0017 (5)
O3	0.0310 (8)	0.0206 (7)	0.0336 (7)	0.0001 (6)	0.0176 (6)	−0.0022 (6)
O4	0.0273 (7)	0.0194 (6)	0.0198 (6)	−0.0003 (5)	0.0036 (5)	−0.0022 (5)
O5	0.0265 (8)	0.0257 (7)	0.0314 (7)	−0.0034 (6)	0.0053 (6)	−0.0094 (6)
O6	0.0400 (10)	0.0393 (9)	0.0388 (9)	−0.0006 (8)	0.0065 (8)	0.0140 (8)
N1	0.0162 (7)	0.0194 (7)	0.0142 (6)	0.0017 (6)	0.0035 (5)	−0.0004 (5)
N2	0.0231 (8)	0.0165 (7)	0.0222 (7)	0.0021 (6)	0.0080 (6)	0.0000 (6)
N3	0.0219 (9)	0.0358 (10)	0.0256 (8)	0.0091 (7)	0.0048 (7)	−0.0014 (7)
N4	0.0683 (15)	0.0228 (9)	0.0382 (11)	−0.0077 (9)	0.0102 (10)	−0.0057 (8)
C1	0.0204 (9)	0.0164 (8)	0.0134 (7)	0.0007 (7)	0.0032 (6)	−0.0038 (6)
C2	0.0172 (8)	0.0170 (8)	0.0141 (7)	0.0002 (7)	0.0031 (6)	−0.0016 (6)
C3	0.0222 (9)	0.0218 (9)	0.0156 (8)	0.0010 (7)	0.0072 (7)	−0.0015 (7)
C4	0.0249 (10)	0.0235 (9)	0.0161 (8)	0.0043 (7)	0.0036 (7)	0.0021 (7)
C5	0.0176 (9)	0.0221 (9)	0.0203 (8)	0.0027 (7)	0.0017 (7)	−0.0051 (7)
C6	0.0189 (9)	0.0268 (9)	0.0207 (8)	−0.0022 (7)	0.0074 (7)	−0.0020 (7)
C7	0.0214 (9)	0.0203 (9)	0.0167 (8)	−0.0016 (7)	0.0048 (7)	0.0010 (6)
C8	0.0166 (9)	0.0202 (9)	0.0213 (8)	0.0039 (7)	0.0026 (7)	−0.0008 (7)
C9	0.0240 (10)	0.0180 (8)	0.0194 (8)	0.0033 (7)	0.0003 (7)	0.0000 (7)
C10	0.0232 (10)	0.0223 (9)	0.0278 (9)	0.0021 (8)	0.0033 (8)	−0.0008 (7)
C11	0.0294 (11)	0.0216 (10)	0.0373 (11)	−0.0022 (8)	0.0050 (9)	0.0027 (8)
C12	0.0376 (12)	0.0219 (10)	0.0272 (10)	0.0044 (9)	−0.0024 (9)	−0.0031 (8)
C13	0.0516 (14)	0.0230 (10)	0.0230 (10)	0.0044 (9)	0.0114 (9)	−0.0005 (8)
C14	0.0381 (12)	0.0209 (9)	0.0246 (9)	0.0031 (8)	0.0100 (8)	0.0016 (7)
C15	0.0177 (9)	0.0158 (8)	0.0177 (8)	0.0011 (7)	0.0045 (7)	−0.0003 (6)
C16	0.0219 (9)	0.0211 (8)	0.0163 (8)	−0.0013 (7)	0.0056 (7)	0.0021 (7)
C17	0.0200 (9)	0.0234 (9)	0.0147 (8)	−0.0027 (7)	0.0067 (7)	−0.0019 (7)
C18	0.0144 (8)	0.0189 (8)	0.0166 (8)	0.0002 (7)	0.0035 (6)	−0.0010 (6)
C19	0.0177 (9)	0.0196 (8)	0.0152 (8)	0.0023 (7)	0.0039 (6)	0.0017 (6)
C20	0.0215 (9)	0.0198 (8)	0.0181 (8)	−0.0012 (7)	0.0098 (7)	−0.0008 (7)
C21	0.0232 (10)	0.0249 (9)	0.0221 (9)	0.0059 (8)	0.0022 (7)	−0.0018 (7)
C22	0.0265 (11)	0.0347 (11)	0.0333 (11)	−0.0057 (9)	0.0036 (9)	−0.0037 (9)
C23	0.0299 (11)	0.0181 (9)	0.0340 (10)	0.0057 (8)	0.0134 (9)	0.0020 (8)
C24	0.0483 (15)	0.0235 (11)	0.0517 (14)	0.0038 (10)	0.0199 (12)	0.0103 (10)
C25	0.0324 (12)	0.0353 (12)	0.0332 (11)	0.0149 (10)	0.0019 (9)	−0.0003 (9)
C26	0.113 (3)	0.0223 (11)	0.0298 (12)	−0.0021 (14)	0.0087 (14)	−0.0038 (9)

### Geometric parameters (Å, °)

**Table:**

Zn1—Zn1^i^	2.9301 (4)	C10—C11	1.383 (3)
Zn1—O1	2.0224 (13)	C10—H10	0.93
Zn1—O2	2.0207 (13)	C11—H11	0.93
Zn1—O4	2.0459 (13)	C12—N4	1.369 (3)
Zn1—N1	2.0516 (14)	C12—C11	1.415 (3)
O1—C1	1.263 (2)	C12—C13	1.396 (3)
O2—C1^i^	1.272 (2)	C13—H13	0.93
O3—Zn1	2.0819 (14)	C14—C13	1.381 (3)
O3—C8	1.264 (2)	C14—H14	0.93
O4—C8^i^	1.276 (2)	C15—C16	1.384 (2)
O5—C20	1.240 (2)	C15—H15	0.93
O6—H61	0.94 (4)	C16—H16	0.93
O6—H62	0.96 (4)	C17—C16	1.384 (3)
N1—C15	1.344 (2)	C17—C18	1.392 (2)
N1—C19	1.343 (2)	C17—H17	0.93
N2—C20	1.336 (2)	C18—C19	1.385 (2)
N2—C21	1.471 (2)	C19—H19	0.93
N2—C23	1.475 (2)	C20—C18	1.505 (2)
N3—H3A	0.86	C21—C22	1.516 (3)
N4—C26	1.447 (3)	C21—H21A	0.97
N4—H4A	0.86	C21—H21B	0.97
C1—O2^i^	1.272 (2)	C22—H22A	0.96
C2—C1	1.480 (2)	C22—H22B	0.96
C2—C3	1.391 (2)	C22—H22C	0.96
C3—H3	0.93	C23—C24	1.515 (3)
C4—C3	1.381 (3)	C23—H23A	0.97
C4—C5	1.407 (3)	C23—H23B	0.97
C4—H4	0.93	C24—H24A	0.96
C5—N3	1.363 (2)	C24—H24B	0.96
C6—C5	1.410 (3)	C24—H24C	0.96
C6—H6	0.93	C25—N3	1.440 (3)
C7—C2	1.403 (2)	C25—H25A	0.96
C7—C6	1.371 (3)	C25—H25B	0.96
C7—H7	0.93	C25—H25C	0.96
C8—O4^i^	1.276 (2)	C26—H26A	0.96
C8—C9	1.479 (2)	C26—H26B	0.96
C9—C10	1.394 (3)	C26—H26C	0.96
C9—C14	1.400 (3)		
			
O1—Zn1—Zn1^i^	73.77 (4)	C12—C11—H11	119.7
O1—Zn1—O3	86.65 (6)	N4—C12—C11	118.8 (2)
O1—Zn1—O4	88.23 (6)	N4—C12—C13	122.8 (2)
O1—Zn1—N1	94.78 (5)	C13—C12—C11	118.43 (19)
O2—Zn1—Zn1^i^	86.29 (4)	C12—C13—H13	119.9
O2—Zn1—O1	159.63 (5)	C14—C13—C12	120.18 (19)
O2—Zn1—O3	90.72 (6)	C14—C13—H13	119.9
O2—Zn1—O4	87.59 (5)	C9—C14—H14	119.2
O2—Zn1—N1	105.52 (5)	C13—C14—C9	121.57 (19)
O3—Zn1—Zn1^i^	70.66 (4)	C13—C14—H14	119.2
O4—Zn1—Zn1^i^	89.93 (4)	N1—C15—C16	122.34 (16)
O4—Zn1—O3	160.59 (5)	N1—C15—H15	118.8
O4—Zn1—N1	106.76 (6)	C16—C15—H15	118.8
N1—Zn1—Zn1^i^	159.67 (4)	C15—C16—H16	120.5
N1—Zn1—O3	92.33 (6)	C17—C16—C15	119.04 (16)
C1—O1—Zn1	135.44 (13)	C17—C16—H16	120.5
C1^i^—O2—Zn1	119.36 (11)	C16—C17—C18	119.00 (16)
C8—O3—Zn1	139.69 (12)	C16—C17—H17	120.5
C8^i^—O4—Zn1	115.95 (12)	C18—C17—H17	120.5
H62—O6—H61	103 (3)	C17—C18—C20	120.11 (15)
C15—N1—Zn1	125.96 (12)	C19—C18—C17	118.48 (16)
C19—N1—Zn1	115.38 (11)	C19—C18—C20	120.80 (15)
C19—N1—C15	118.45 (15)	N1—C19—C18	122.65 (16)
C20—N2—C21	124.58 (15)	N1—C19—H19	118.7
C20—N2—C23	118.32 (16)	C18—C19—H19	118.7
C21—N2—C23	117.04 (16)	O5—C20—N2	122.74 (17)
C5—N3—C25	122.12 (17)	O5—C20—C18	117.80 (17)
C5—N3—H3A	118.9	N2—C20—C18	119.45 (16)
C25—N3—H3A	118.9	N2—C21—C22	111.97 (16)
C12—N4—C26	123.4 (2)	N2—C21—H21A	109.2
C12—N4—H4A	118.3	N2—C21—H21B	109.2
C26—N4—H4A	118.3	C22—C21—H21A	109.2
O1—C1—O2^i^	124.19 (17)	C22—C21—H21B	109.2
O1—C1—C2	117.03 (15)	H21A—C21—H21B	107.9
O2^i^—C1—C2	118.78 (15)	C21—C22—H22A	109.5
C3—C2—C1	122.21 (16)	C21—C22—H22B	109.5
C3—C2—C7	118.19 (16)	C21—C22—H22C	109.5
C7—C2—C1	119.55 (15)	H22A—C22—H22B	109.5
C2—C3—H3	119.5	H22A—C22—H22C	109.5
C4—C3—C2	121.08 (17)	H22B—C22—H22C	109.5
C4—C3—H3	119.5	N2—C23—C24	111.91 (16)
C3—C4—C5	120.62 (17)	N2—C23—H23A	109.2
C3—C4—H4	119.7	N2—C23—H23B	109.2
C5—C4—H4	119.7	C24—C23—H23A	109.2
N3—C5—C4	121.78 (17)	C24—C23—H23B	109.2
N3—C5—C6	119.95 (17)	H23A—C23—H23B	107.9
C4—C5—C6	118.26 (17)	C23—C24—H24A	109.5
C5—C6—H6	119.9	C23—C24—H24B	109.5
C7—C6—C5	120.25 (17)	C23—C24—H24C	109.5
C7—C6—H6	119.9	H24A—C24—H24B	109.5
C2—C7—H7	119.2	H24A—C24—H24C	109.5
C6—C7—C2	121.56 (17)	H24B—C24—H24C	109.5
C6—C7—H7	119.2	N3—C25—H25A	109.5
O3—C8—O4^i^	123.76 (17)	N3—C25—H25B	109.5
O3—C8—C9	117.69 (16)	N3—C25—H25C	109.5
O4^i^—C8—C9	118.55 (16)	H25A—C25—H25B	109.5
C10—C9—C8	121.59 (17)	H25A—C25—H25C	109.5
C10—C9—C14	118.31 (18)	H25B—C25—H25C	109.5
C14—C9—C8	120.10 (17)	N4—C26—H26A	109.5
C9—C10—H10	119.6	N4—C26—H26B	109.5
C11—C10—C9	120.71 (19)	N4—C26—H26C	109.5
C11—C10—H10	119.6	H26A—C26—H26B	109.5
C10—C11—C12	120.6 (2)	H26A—C26—H26C	109.5
C10—C11—H11	119.7	H26B—C26—H26C	109.5
			
Zn1^i^—Zn1—O1—C1	−10.58 (16)	C20—N2—C23—C24	86.7 (2)
O2—Zn1—O1—C1	−22.8 (3)	C21—N2—C23—C24	−96.1 (2)
O3—Zn1—O1—C1	60.23 (17)	C3—C2—C1—O1	175.07 (16)
O4—Zn1—O1—C1	−101.03 (17)	C3—C2—C1—O2^i^	−4.9 (3)
N1—Zn1—O1—C1	152.30 (17)	C7—C2—C1—O1	−2.3 (2)
Zn1^i^—Zn1—O2—C1^i^	1.39 (12)	C7—C2—C1—O2^i^	177.77 (16)
O1—Zn1—O2—C1^i^	13.1 (2)	C1—C2—C3—C4	−176.37 (17)
O3—Zn1—O2—C1^i^	−69.17 (13)	C7—C2—C3—C4	1.0 (3)
O4—Zn1—O2—C1^i^	91.48 (13)	C3—C4—C5—N3	177.78 (18)
N1—Zn1—O2—C1^i^	−161.79 (12)	C5—C4—C3—C2	−0.3 (3)
Zn1^i^—Zn1—O4—C8^i^	−0.04 (12)	C3—C4—C5—C6	−1.3 (3)
O1—Zn1—O4—C8^i^	73.72 (13)	C4—C5—N3—C25	5.1 (3)
O2—Zn1—O4—C8^i^	−86.33 (13)	C6—C5—N3—C25	−175.77 (19)
O3—Zn1—O4—C8^i^	−1.0 (2)	C7—C6—C5—N3	−176.90 (18)
N1—Zn1—O4—C8^i^	168.18 (12)	C7—C6—C5—C4	2.2 (3)
Zn1^i^—Zn1—N1—C15	−165.91 (10)	C6—C7—C2—C1	177.35 (17)
Zn1^i^—Zn1—N1—C19	8.7 (2)	C6—C7—C2—C3	−0.1 (3)
O1—Zn1—N1—C15	139.65 (15)	C2—C7—C6—C5	−1.5 (3)
O1—Zn1—N1—C19	−45.76 (13)	O3—C8—C9—C10	163.73 (18)
O2—Zn1—N1—C15	−42.11 (16)	O3—C8—C9—C14	−15.5 (3)
O2—Zn1—N1—C19	132.48 (12)	O4^i^—C8—C9—C10	−15.9 (3)
O3—Zn1—N1—C15	−133.51 (15)	O4^i^—C8—C9—C14	164.81 (18)
O3—Zn1—N1—C19	41.08 (13)	C8—C9—C10—C11	−175.97 (18)
O4—Zn1—N1—C15	50.06 (15)	C14—C9—C10—C11	3.3 (3)
O4—Zn1—N1—C19	−135.35 (12)	C8—C9—C14—C13	177.63 (19)
Zn1—O1—C1—O2^i^	13.4 (3)	C10—C9—C14—C13	−1.6 (3)
Zn1—O1—C1—C2	−166.52 (12)	C9—C10—C11—C12	−1.1 (3)
C8—O3—Zn1—Zn1^i^	1.03 (19)	C13—C12—N4—C26	−0.3 (4)
C8—O3—Zn1—O1	−72.9 (2)	C11—C12—N4—C26	−180.0 (2)
C8—O3—Zn1—O2	86.8 (2)	N4—C12—C11—C10	176.8 (2)
C8—O3—Zn1—O4	2.1 (3)	C13—C12—C11—C10	−2.9 (3)
C8—O3—Zn1—N1	−167.6 (2)	N4—C12—C13—C14	−175.2 (2)
Zn1—O3—C8—O4^i^	−1.4 (3)	C11—C12—C13—C14	4.5 (3)
Zn1—O3—C8—C9	178.98 (14)	C9—C14—C13—C12	−2.3 (3)
Zn1—N1—C15—C16	174.78 (13)	N1—C15—C16—C17	−1.9 (3)
C19—N1—C15—C16	0.3 (3)	C18—C17—C16—C15	1.9 (3)
Zn1—N1—C19—C18	−173.78 (14)	C16—C17—C18—C19	−0.4 (3)
C15—N1—C19—C18	1.2 (3)	C16—C17—C18—C20	−171.52 (17)
C21—N2—C20—O5	−176.81 (17)	C17—C18—C19—N1	−1.2 (3)
C21—N2—C20—C18	3.5 (3)	C20—C18—C19—N1	169.86 (16)
C23—N2—C20—O5	0.2 (3)	O5—C20—C18—C17	67.7 (2)
C23—N2—C20—C18	−179.51 (15)	O5—C20—C18—C19	−103.2 (2)
C20—N2—C21—C22	97.4 (2)	N2—C20—C18—C17	−112.63 (19)
C23—N2—C21—C22	−79.6 (2)	N2—C20—C18—C19	76.5 (2)

Symmetry codes: (i) −*x*, −*y*, −*z*.

### Hydrogen-bond geometry (Å, °)

**Table:**

*D*—H···*A*	*D*—H	H···*A*	*D*···*A*	*D*—H···*A*
N3—H3A···O6^ii^	0.86	2.50	3.105 (3)	128
N4—H4A···O6^iii^	0.86	2.07	2.922 (3)	171
O6—H61···O4^iv^	0.93 (4)	2.07 (4)	2.875 (2)	143 (3)
O6—H61···O2^iv^	0.93 (4)	2.37 (4)	3.117 (2)	137 (3)
O6—H62···O5^v^	0.96 (4)	1.81 (4)	2.741 (2)	162 (3)

Symmetry codes: (ii) *x*−2, *y*, *z*−1; (iii) *x*−1/2, −*y*+1/2, *z*−1/2; (iv) −*x*+1, −*y*, −*z*+1; (v) *x*+1, *y*, *z*.
